# Influence of Mechanical Ventilation Modes on the Efficacy of Nebulized Bronchodilators in the Treatment of Intubated Adult Patients with Obstructive Pulmonary Disease

**DOI:** 10.3390/pharmaceutics15051466

**Published:** 2023-05-11

**Authors:** Cibelle Andrade Lima, Shirley Lima Campos, Monique Pontes Bandeira, Wagner Souza Leite, Daniella Cunha Brandão, Juliana Fernandes, James B. Fink, Armele Dornelas de Andrade

**Affiliations:** 1 Physiotherapy Depatment, Universidade Federal do Rio Grande do Norte, Natal 59078-970, RN, Brazil; 2 Physiotherapy Depatment, Universidade Federal de Pernambuco, Recife 50740–560, PE, Brazil; 3 Department of Cardiopulmonary Science, Division of Respiratory, CA Rush University Medical Center, Chicago, IL 60612, USA; 4 Aerogen Pharma, San Mateo, CA 94402, USA

**Keywords:** bronchodilator, mechanical ventilation, asthma, COPD, nebulizers and vaporizers, inhalation

## Abstract

Background: Little has been reported in terms of clinical outcomes to confirm the benefits of nebulized bronchodilators during mechanical ventilation (MV). Electrical Impedance Tomography (EIT) could be a valuable method to elucidate this gap. Objective: The purpose of this study is to evaluate the impact of nebulized bronchodilators during invasive MV with EIT by comparing three ventilation modes on the overall and regional lung ventilation and aeration in critically ill patients with obstructive pulmonary disease. Method: A blind clinical trial in which eligible patients underwent nebulization with salbutamol sulfate (5 mg/1 mL) and ipratropium bromide (0.5 mg/2 mL) in the ventilation mode they were receiving. EIT evaluation was performed before and after the intervention. A joint and stratified analysis into ventilation mode groups was performed, with *p* < 0.05. Results: Five of nineteen procedures occurred in controlled MV mode, seven in assisted mode and seven in spontaneous mode. In the intra-group analysis, the nebulization increased total ventilation in controlled (*p* = 0.04 and ⅆ = 2) and spontaneous (*p* = 0.01 and ⅆ = 1.5) MV modes. There was an increase in the dependent pulmonary region in assisted mode (*p* = 0.01 and ⅆ = 0.3) and in spontaneous mode (*p* = 0.02 and ⅆ = 1.6). There was no difference in the intergroup analysis. Conclusions: Nebulized bronchodilators reduce the aeration of non-dependent pulmonary regions and increase overall lung ventilation but there was no difference between the ventilation modes. As a limitation, it is important to note that the muscular effort in PSV and A/C PCV modes influences the impedance variation, and consequently the aeration and ventilation values. Thus, future studies are needed to evaluate this effort as well as the time on ventilator, time in UCI and other variables.

## 1. Introduction

Inhaled bronchodilator drug administration during invasive mechanical ventilation (MV) is a common practice in the Intensive Care Unit (ICU). The effectiveness of inhalation therapy during invasive MV is largely dependent on factors related to the inhalation device, device placement, ventilator circuit, MV mode and parameters. These factors influence the ventilatory pattern, aerosol particle size, and hygroscopicity and are directly linked to the ability to penetrate the airways and be deposited in the lungs [1,2,3,4].

Little has been reported in terms of clinical response and outcomes to confirm the aerosol therapy benefits identified by in vivo and in vitro studies. Despite airway resistance, compliance and lung deposition by scintigraphy analysis with radioisotopes having shown improvement after inhaler therapy, they do not provide markers to assess clinical therapeutic responses to inhaled drugs, but rather mechanical markers or the bioavailability of radioactive markers in the lungs [5,6,7,8,9,10]. Electrical Impedance Tomography (EIT) could be a valuable method to elucidate this gap. EIT is an innovative method, gaining acceptance to evaluate pulmonary ventilation and aeration in critically ill patients, including monitoring pulmonary responses to therapeutic measures, such as nebulized bronchodilators [2,11,12,13].

Aerosol is commonly administered during invasive MV, with various modes including controlled modes such as Pressure Control Ventilation (PCV), Assist/Control such as Pressure Assist Control Ventilation (A/C PCV) and spontaneous breathing with Pressure Support Ventilation (PSV). All breathing in PCV mode is initiated by the machine; in contrast, A/C PCV mode can be initiated by the machine or the patient muscle effort. Time is the cycle in both cases. PSV is a spontaneous mode and has all breathing initiated by the patient’s effort and has a flow cycle [6,9].

As the pulmonary deposition of the aerosol is influenced by the ventilatory pattern and previous studies with computed tomography and EIT show that there are differences in ventilation distribution between the MV modes according to the proportion of ventilatory assistance and respiratory work developed by the patient, we can speculate that there are differences in ventilation and aeration between MV modes after bronchodilator aerosol therapy [14,15,16].

Furthermore, previous in vitro studies have shown that spontaneous modes, such as continuous positive airway pressure (CPAP), increase pulmonary aerosol deposition by more than 30% when compared to controlled ventilation modes under equal tidal volume conditions [2,5]. So, we hypothesize that the spontaneous mode with Pressure Support Ventilation yields a better clinical response to aerosol bronchodilators in aeration and ventilation compared to the controlled and assisted modes.

This study aims to evaluate the impact of nebulized bronchodilators during invasive MV with EIT by comparing three ventilation modes on the overall and regional lung ventilation and aeration in critically ill patients with obstructive pulmonary disease.

## 2. Materials and Methods

This is a blinded clinical trial developed in the intensive care unit of Recife, located in the state of Pernambuco, Brazil. The study included patients with obstructive pulmonary disease, intubated, receiving invasive MV and prescribed to receive nebulization with bronchodilator drugs. Subjects were excluded if they: were receiving intravenous bronchodilators, were experiencing hemodynamic instability (mean blood pressure < 65 mmHg), had airway resistance less than 13 cm H_2_O, had an undrained pneumothorax, bronchopleural or tracheoesophageal fistula, granulomas, tracheal stenosis, chest trauma, cardiopathy or some contraindication for MV disconnection, an endotracheal tube inner diameter less than 7.5 mm, tracheal hypersecretivity, or an air leak around the endotracheal tube despite adjusting the cuff pressure.

Sample size was calculated using a software program developed by the Mallinckrodt MGH General Clinical Research Center (Boston, MA, USA) based on the results of the first 10 patients collected. The statistical power to detect difference was 80% and the significance level was 0.05. The sample size of *n* = 16 patients was found considering that the minimum detectable difference of the main outcome variable of the Electric Impedance Tomography (∆Z_TOT_) before and after the bronchodilator nebulization procedure was 0.17 and the standard deviation was 0.16.

The study was approved by the Institutional Research Ethics Committee with identification number: 63837617.4.0000.5198 *n* and registered on ClinicalTrials.gov with identifier code: NCT03271905.

### 2.1. Procedures

Anthropometric and clinical data were collected from the patients. A bedside assessment was conducted to identify the use of vasoactive drugs, sedation, artificial airway (type, size, and position), humidification, ventilatory pattern, thoracic expansibility, and pulmonary auscultation. The Richmond Agitation-Sedation Scale (RASS) was accessed, and the score was: +4 for combative patients, +3 very agitated, +2 agitated, +1 restless, 0 for alert and calm patients, −1 drowsy, −2 light sedation, −3 moderate sedation, −4 deep sedation and −5 for unarousable. The cuff pressure was measured using a cuff pressure gauge (VBM Medzintechnik GmbH, Sulz, Germany) to adjust the minimum volume of tracheal occlusion when necessary, maintaining a minimum pressure of 25 cmH_2_O. An open tracheal aspiration was performed if the patient presented any sign of mucus accumulation or obstruction, and then the protocol was started after a wait of 30 min.

**Figure 1:**
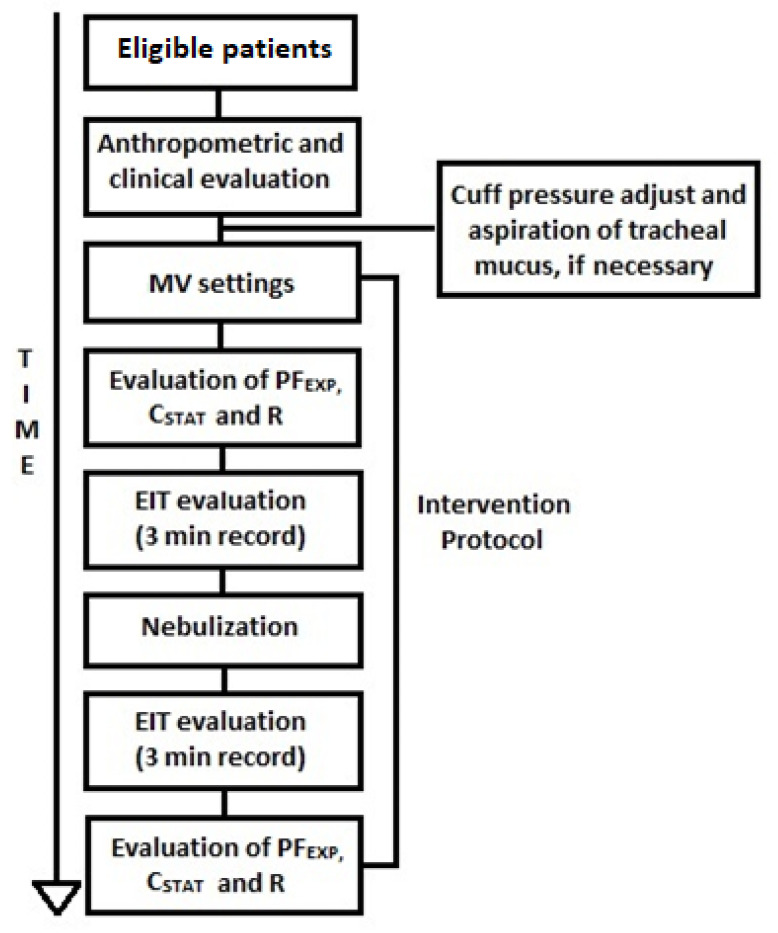
Method flowchart. MV: Mechanical Ventilation, PF_EXP_: Peak Expiratory Flow, C_STAT_: Static Compliance, R: Airway Resistance, EIT: Electrical Impedance Tomography, min: minute.

The intervention protocol consisted of administering ordered bronchodilator nebulization associated with invasive MV. The ventilation mode the patient was receiving prior to study initiation was maintained, but adjustments (described below) were made with the objective of optimizing aerosol deposition in that mode. Patients were evaluated through EIT before and immediately after nebulization. Ventilator assessment data were also collected, including Peak Expiratory Flow (PF_EXP_), Static Compliance (C_STAT_), and airway resistance (R) (Figure 1). A patient could receive a new intervention protocol post-intervention if they were to receive another ventilation mode.

### 2.2. Nebulization Protocol

Salbutamol sulfate (Aerolin inhalation, 5 mg/1 mL) and ipratropium bromide (Atrovent, 0.5 mg/2 mL) were diluted in a saline solution (saline 0.9%) to a fill volume of 4 mL. The equipment used was a MESH nebulizer (Aerogen Solo: Aerogen Ltd., Galway, Ireland) placed in the inspiratory limb of the ventilator circuit immediately before the “Y” piece using a 22 mm T adapter [17]. The MESH nebulizer was continuously operated by an electric controller connected to the main power and produced aerosol with an average particle size of <5 μm (per manufacturer label). Each dose continued until no aerosol was generated.

The heat moisture exchanger (HME) used during MV was removed during nebulization and replaced after the end of the complete intervention protocol.

### 2.3. Ventilator Parameters

Patients receiving controlled or assisted-controlled ventilation modes with Pressure Control Ventilation (PCV) had the parameters adjusted to a tidal volume (V_T_) of 6 mL/kg of ideal weight, and peak inspiratory pressure (PIP) limit of 35 cmH_2_O. Inspiratory time (Ti) and respiratory rate (RR) were adjusted to maintain Ti/T_TOT_ (Duty cycle) > 0.3, without causing dynamic autoPEEP [1,6,9,18,19,20,21,22,23].

The patient controls the Ti, T_TOT_, RR and inspiratory flow in spontaneous ventilation with Pressure Support Ventilation (PSV), so that only the Δ pressure from baseline (ΔP) was adjusted to maintain the V_T_ at 6 mL/kg of ideal weight. The end of the inspiratory phase was determined when flow decreased to 25% of the patient peak inspiratory flow [1,24].

Sedated patients in controlled PC mode had PEEP adjusted to 80% of the patient’s previously evaluated static autoPEEP. The PEEP setting for patients on assist-controlled and spontaneous ventilation modes was between 5 and 8 cm H_2_O, in order to avoid dynamic autoPEEP as determined via graphic ventilator monitoring [25].

The pre-dose data collection only started after adjusting the ventilator parameters, and there was an acclimatization period by the patient. The ventilator settings were maintained during nebulization and until the end of all data collection after the procedure.

### 2.4. Pulmonary Mechanics

Static Compliance (C_STAT_), airway resistance (R) and Peak Expiratory Flow (PF_EXP_) were assessed before and immediately after the nebulization procedure. The evaluation of C_STAT_ and R was only possible in sedated patients who were in controlled mechanical ventilation. Thus, an inspiratory pause of 3 to 5 s was performed to identify the peak inspiratory pressure (PIP) and plateau pressure (P_PLAT_) and supply the C_STAT_ and R data in the ventilator monitor. The PF_EXP_ value was collected through the ventilator monitor in all ventilation modes.

### 2.5. Electrical Impedance Tomography

An impedance tomography device (ENLIGHT, Timpel, Brazil) was used with the patient in supine position in a bed elevated to 30°, and a belt with 32 electrodes was applied to their chest in the position corresponding to the 4th–5th intercostal space on shaved skin. The electrodes were positioned for reading electrocardiograms and a flow sensor was attached to the endotracheal tube. After these procedures, the data were recorded for 3 min before and immediately after nebulization [26,27].

For EIT image acquisition, small/harmless electrical currents (5–8 nA, 125 KHz) are emitted through electrode pairs in rotating sequence and the potential differences are captured in the other receiving electrode. Variations in thoracic impedance were evaluated through the LabView software program (National Instruments, Austin, TX, USA) and the data were recorded in separate files for each patient for further analysis. One minute of the most stable acquisition (3000 frames) was chosen to analyze the variables. The outcome variables were: total lung ventilation by total impedance variation of tidal volume (ΔZ_TOT_); total lung aeration, which reflects the functional residual capacity trough end-expiratory lung volume (EELV_TOT_); and the distribution of regional lung ventilation and aeration by impedance variation in the region of interest (ROI). The analyzed ROIs were the dependent and non-dependent pulmonary region, as well as the lower and upper half of the lung in relation to gravity, respectively.

### 2.6. Statistical Analysis

Statistical analysis was conducted using the SPSS 20.0 statistical software program, with a 95% confidence interval for all the variables. The Shapiro–Wilk test was applied to verify normality and the Levene’s test was used for homogeneity of variances.

The clinical characteristics of patients are presented as mean ± standard deviation for quantitative variables and as absolute and relative frequencies for qualitative variables. The Chi-squared and Kruskal–Wallis tests were performed to compare differences between groups. The Wilcoxon test was applied to compare the EIT and pulmonary mechanic variables pre- and post-intervention in each group. The intergroup analysis was performed by the Kruskal–Wallis test.

Cohen’s d effect sizes were calculated using the variation obtained in intra- and intergroup comparison according to ventilation modes. The Effect Size Calculator by Lenhard and Lenhard (2016) “https://www.psychometrica.de/effect_size.html” (accessed on 22 April 2019) was used, and a Cohen’s d effect size greater than 0.8 was considered large, 0.5 was moderate, and 0.2 was small [28,29].

## 3. Results

**Figure 2:**
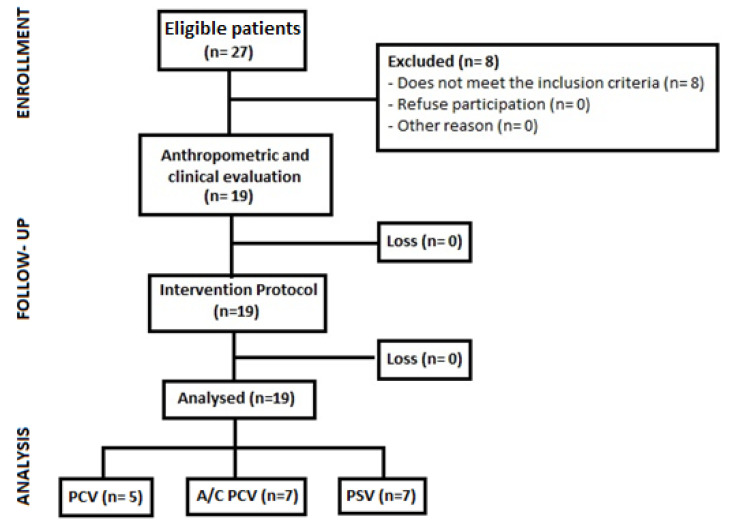
Patient recruitment and follow-up flowchart. PCV: pressure-controlled ventilation, A/C PCV: assist-control pressure ventilation, PSV: spontaneous pressure support ventilation.

A total of 27 screened patients were eligible according to the inclusion criteria. The final sample consisted of 19 patients stratified into 3 ventilation modes: Pressure Control Ventilation (PCV), Assist/Control Pressure Ventilation (A/C PCV) and Pressure Support Ventilation (PSV). Five patients were excluded for hemodynamic instability, two for associated cardiac conditions and one for an endotracheal tube diameter less than 7.5 mm (Figure 2).

Anthropometric and clinical data of participants are shown in Table 1. Respiratory infection (RI) was the primary cause of the exacerbation of the underlying lung disease in all cases, resulting in acute respiratory failure and MV. The RI in three of these cases (one in each ventilation mode group) was of secondary origin (nosocomial pneumonia) due to hospitalization for orthopedic trauma. Two cases of RI, both in COPD patients, progressed to sepsis—one in A/C PCV and one in the PSV group. Humidification was provided with heat and moisture exchangers (HMEs) for all patients.

### 3.1. Analysis of the Nebulized Bronchodilator Effect during Invasive MV

**Figure 3:**
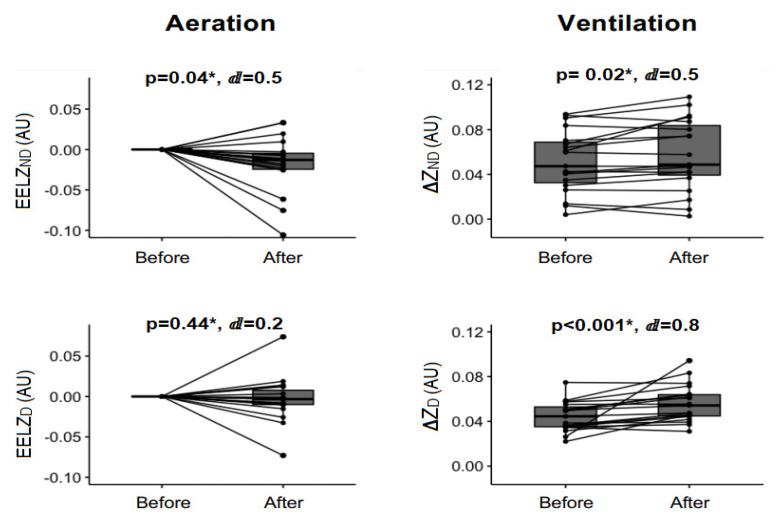
Ventilation and aeration EIT analysis. AU: arbitrary unit, ∆ZND: impedance variation on non-dependent pulmonary region, ∆ZD: impedance variation on dependent pulmonary region, EELVND: end-expiratory lung volume on non-dependent pulmonary region, EELVD: end-expiratory lung volume on dependent pulmonary region. * Wilcoxon signed rank test. ⅆ: value of Cohen’s d.

Figure 3 shows a pre-post joint analysis of all ventilation modes. The nebulized bronchodilator increased the total impedance ventilation ΔZ_TOT_ from 0.09 (0.036) to 0.113 (0.034), reflecting an increase of 25.5% in ventilation, with *p* < 0.01 and a large effect size (ⅆ = 0.8). In the ROI analysis, there was an increase in non-dependent impedance variation (ΔZ_ND_) and in dependent impedance variation (ΔZ_D_) lung regions, with *p* = 0.02, ⅆ = 0.5 and *p* < 0.01, ⅆ = 0.8, respectively.

The decrease in the total end-expiratory lung volume (EELV_TOT_) from −0.0103 (0.0024) to −0.0308 (0.0037) shows a significant reduction in total lung aeration after the nebulization procedure (*p* = 0.02, ⅆ = 0.5). There was a significant reduction only in the non-dependent pulmonary region end-expiratory lung volume (EELV_ND_) (*p* = 0.04 and ⅆ = 0.5) in the regional aeration analysis.

There was an increase in tidal volume (V_T_) evaluated by the EIT sensor flow from 0.339 (0.098) to 0.478 (0.167) (*p* < 0.01 and ⅆ = 0.65), but there was no statistical difference in minute volume (V_E_) and respiratory rate (RR) after the procedure when compared to baseline values.

There was a reduction in R in the ventilator assessment from 15.50 (2.39) to 11.40 (2.34) (*p* = 0.01 and ⅆ = 0.6), and an increase in PF_EXP_ from 41.75 (14.98) to 48.75 (16.42) (*p* < 0.01 and ⅆ = 0.7) after bronchodilator nebulization, both with a moderate effect size. There was no change in C_STAT_.

### 3.2. Analysis of the Nebulized Bronchodilator Effect during Different Ventilation Modes

A stratified analysis of ventilation and aeration was performed in three groups according to ventilation modes: PCV, A/C PCV and PSV. Table 2 shows a similar difference between groups in the baseline values of EIT variables, so the patients were comparable in ventilation, aeration, tidal volume, and inspiratory time conditions. All aeration variables had basal values normalized to 0.

**Figure 4:**
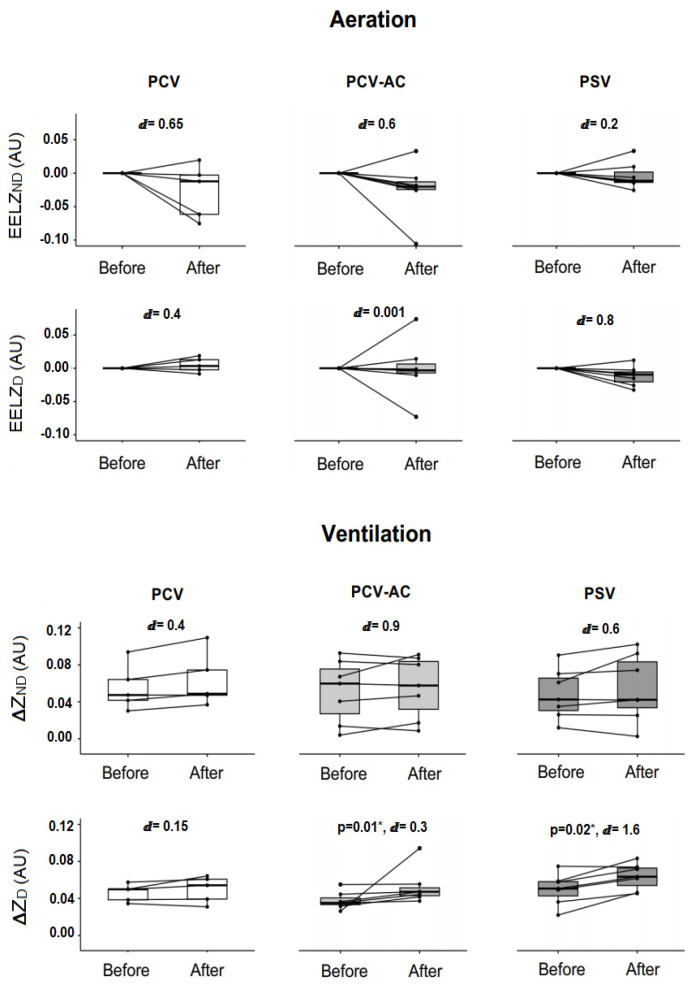
Intra-group analysis of regional and global pulmonary ventilation and aeration. AU: arbitrary unit, PCV: pressure-controlled ventilation, A/C PCV: assisted-controlled pressure ventilation, PSV: pressure support ventilation, ∆Z_ND_: impedance variation in non-dependent pulmonary region, ∆Z_D_: impedance variation in dependent pulmonary region, ∆Z _TOT_: total impedance variation, EELV_ND_: end-expiratory lung volume in non-dependent pulmonary region, EELV_D_: end-expiratory lung volume in dependent pulmonary region, EELV_TOT_: total end-expiratory lung volume, Pre: before nebulization, Post: immediately after nebulization. * Wilcoxon test. ⅆ: value of Cohen’s d.

The PCV group had a score of −5 on the Richmond Agitation-Sedation Scale (RASS). In the intra-group analysis, the PCV mode increased total ventilation after nebulization (*p* = 0.04, ⅆ = 0.2) with no major increase in any of the evaluated regions of interest (ΔZ_D_ and ΔZ_ND_). There was no significant difference in the overall and regional EELV values, but the reduction in total aeration and in the non-dependent pulmonary region after nebulization had a moderate effect size of ⅆ = 0.7 and 0.65, respectively (Figure 4).

The A/C PCV group had a RASS score ranging from −3 to −1. The total value of ventilation, ΔZ_TOT_, increases with a large effect size of ⅆ = 1.1; however, there were no significant differences in *p*-value aeration and ventilation between pre- and post-nebulization. In the intra-group analysis of the ROIs, the mean increase in ΔZ_ND_ and decrease in EELV_ND_ had a large and a moderate effect size of ⅆ = 0.9 and ⅆ = 0.6, respectively, instead of a non-significant *p*-value. In contrast, there was an increase in ΔZ_D_ after nebulization (*p* = 0.018) with ⅆ = 0.3, and no change in EELV_D_.

The PSV group (RASS ranging from −2 to +1) had a significant increase in total ventilation and in the dependent pulmonary region after nebulization (*p* = 0.01 and *p* = 0.02, respectively), both with a large Cohen’s d (Figure 4). Figure 4 also shows that there was no difference in the *p*-value of the intra-group aeration analysis, but the decrease that occurred in EELV_D_ achieved a large effect size (ⅆ = 0.8).

There was no difference in the total and regional ventilation and aeration in the intergroup analysis. The effect sizes were small in all variables.

## 4. Discussion

### 4.1. Effects of Nebulized Bronchodilator during Invasive MV

The main effects of bronchodilator nebulization in obstructive critically ill patients during invasive MV are:

- A decrease in total lung aeration and an increase in total lung ventilation;
- A decrease in airway resistance and a consequent increase in tidal volume and peak expiratory flow.

This study is a pioneer in evaluating clinical outcomes such as overall and regional pulmonary ventilation and aeration. The EIT is a simple non-invasive and non-radiation emission method that enabled the evaluation of relevant pulmonary responses such as pulmonary ventilation and aeration through changes in conductivity of the tissues, as well as inhaled bronchodilator therapy during invasive MV in critically ill patients [12,13,30].

The obtained results provide clinical support to confirm the benefits of aerosolized bronchodilators in obstructive critically ill patients (both in ventilator-dependent and in spontaneously breathing patients with artificial airways), as previously described in in vitro studies [5,6,7,17,19,21].

However, the pulmonary deposition of radioaerosols in mechanically ventilated individuals, as found in the results in studies by O’Riordan et al. (1999) and Dugernier (2017), corroborates the reduction in total end-expiratory lung volume (air trapping), the increase in overall ventilation, and other clinical benefits found in this study. Furthermore, two studies using a chromatographic assay in urine excretion observed a good pulmonary bioavailability of nebulized albuterol and ipratropium in ventilated individuals, which explains the good response of these nebulized drugs in our clinical markers [31,32].

In line with the reduction in air trapping and the improvement in ventilation, there was also a significant increase in the tidal volume (V_T_) post-procedure. This finding is directly related to the reduction in airway resistance and consequent increase in expiratory flow, both constituting clinical outcomes that have already been documented in previous studies [33,34,35]. All these results reveal that the pulmonary deposition of the bronchodilator was sufficient to produce the aerosol effect: smooth muscle relaxation in the airway and reversion of the obstructive pattern [36,37].

We observed that the increase in V_T_ was not followed by an increase in the V_E_. This is probably due to variations in the RR of the patients in assisted and spontaneous ventilation modes. The relief of airflow obstruction with the administration of bronchodilators reduces respiratory work and RR in patients with preserved neural drive, as demonstrated in the study by Galindo-Filho et al. (2013) [38].

ROI analysis showed that aeration reduction after nebulization was significant in the non-dependent pulmonary region, and the increase in ventilation occurred in both the non-dependent and dependent pulmonary regions analyzed. This can be explained in part by regional ventilation differences in mechanically ventilated patients. The alveoli of the non-dependent pulmonary region are more aerated, less compliant, and less likely to ventilate in the supine position, but differently from physiological breathing, the positive pressure in MV tends to direct airflow to the region with lower resistance—the non-dependent pulmonary region. Thus, it is possible that a greater bronchodilator drug deposition occurred in this area, decreasing aeration and air trapping, causing the increase in the non-dependent pulmonary ventilation [15,39,40].

In contrast, the alveoli of the pulmonary dependent regions are poorly aerated and more compliant, so despite the reduction of diaphragm muscle activity during MV—depending on the sedation level and ventilation mode of the patient—the dependent lung area is more likely to increase ventilation in changes of airway resistance [41,42].

### 4.2. Effects of Nebulized Bronchodilator in Different Ventilation Modes

Although there was no difference in the intergroup analysis of PCV, A/C PCV and PSV ventilation modes, the intra-group analysis shows important results to be discussed:

- There was an increase in total lung ventilation after nebulization in PCV mode with a large effect size, but there was no difference in ROI analysis when compared to baseline values.
- There was a significant increase in dependent pulmonary region ventilation after nebulization in the A/C PCV mode, but the magnitude of this difference given by effect size was small. On the other hand, the increase that occurred in total and non-dependent pulmonary region ventilation had a large effect size, instead of the non-significant *p*-value.
- There was an increase in total lung ventilation and in the pulmonary dependent region after using the nebulizer bronchodilator in PSV mode, both with a large effect size.
- Even without a statistically significant *p*-value, it is important to note that there was a decreasing trend of total aeration with a moderate Cohen’s d in PCV and PSV modes. Additionally, there was a predominant decrease and a large effect size in dependent ROI in the spontaneous mode, while the decrease in controlled and assisted modes was predominant in the non-dependent lung region, with a moderate Cohen’s d.

There is no superiority of any ventilation mode for the efficacy of nebulization, but the three types considered, with breathing being delivered by a mechanical ventilator (controlled, assisted and spontaneous), presented different responses in pulmonary ventilation and aeration after bronchodilator nebulization.

In control ventilation mode (PCV), the machine determines all phases of the respiratory cycle, the respiratory rate, and the inspiratory time, so the patient does not work. When the diaphragm does not act in the breathing process, the air tends to be preferentially directed to the non-dependent lung region. Thus, it is possible that the pulmonary deposition of the bronchodilator is greater in the non-dependent lung region. The decrease in aeration and the increase in ventilation of the non-dependent area had a greater effect size compared to dependent region, demonstrating the major contribution of this region to the significant increase in total ventilation after nebulization [40,41,43].

In the Assist/Control ventilation mode (A/C PCV), a muscle effort is necessary to trigger the ventilator and initiate a respiratory cycle, but the machine still determines the rate and inspiratory time. Therefore, the breathing work is shared between the ventilator and the patient. The effort level that the patient develops may be minimal if all the patient does is trigger the breathing, or it can be considerable if a small pressure application on the ventilator is sufficient to maintain an adequate tidal volume. This leads to different breathing delivery and aerosol deposition patterns, depending on the muscular effort that the patient can exert [43,44,45].

Thus, the magnitude of the decrease in aeration and increase in ventilation after nebulization in the non-dependent pulmonary region suggests that the airflow direction and aerosol deposition had more influence of the positive pressure provided by the ventilator machine than the respiratory muscles. However, the ease of dependent pulmonary alveoli increasing ventilation in changes of pulmonary mechanics, such as the airway reduction favored by the bronchodilator, allied to some diaphragmatic muscle activity, also explains the significant ventilation increase in the dependent lung ROI [39,41].

Furthermore, there was no increase in total ventilation after nebulization in Assist/Control PCV from the changes in ROI ventilation and aeration. Our perception is that this particular MV mode had more patient–ventilator asynchrony, which may have impacted the aerosol deposition and consequently the potential clinical effect. However, as already shown by Thille et al. (2006) [46], we can accept that this factor is inherent to the ventilation mode itself, since adjustments were made to satisfactorily adapt the patient to the ventilator before the intervention protocol.

In a spontaneous mode such as PSV, the patient must generate a muscular effort that is able to actively control the ventilation cycles. In other words, the MV mode produces a higher degree of diaphragmatic muscular activity and therefore the airflow and aerosol tend to be directed to the dependent pulmonary region. This explains the increase in ventilation on the dependent pulmonary region after using the nebulized bronchodilator, which was also sufficient to increase total ventilation, and had a large effect size in both cases.

It is important to note that there was a change in the graphical behavior of the aeration on the dependent ROI in spontaneous mode, which was a decrease of a large magnitude in the difference between pre- and post-nebulization values and was different from the controlled and assisted MV modes. The decrease in total aeration and in the dependent pulmonary region suggest that the reduction in hyperinflation increased total and dependent pulmonary region ventilation [16,47].

### 4.3. Limitations

This study had some limitations. The number of subjects in each mode of ventilation is relatively small, suggesting that a larger population may demonstrate more significance in the areas of trending changes observed. It is important to note that the muscular effort in PSV and A/C PCV modes influences the impedance variation, and consequently the aeration and ventilation values. However, we believe that the difference found in our results is solely due to effect of the bronchodilator, since the patient was in the same ventilation mode and in the same clinical conditions before and after nebulization. Additionally, saline solution at high concentrations alters electrical conductivity and may lead to a “false reduction” in aeration values. However, the saline solution used in our study was minimal and the aeration decreases found in our study were followed by increased ventilation, proving that the use of the bronchodilator in fact changed the pulmonary mechanics, ventilation, and aeration.

## 5. Conclusions

This clinical study supports previous in vitro research and confirms the hypothesis that nebulized bronchodilators reduce the aeration of non-dependent pulmonary regions and increase overall lung ventilation. The bronchodilator was also effective in reducing airway resistance and consequently increasing the tidal volume.

There was no difference in the intergroup analysis of ventilation modes. Therefore, the hypothesis that the spontaneous mode has a better clinical response in aeration and ventilation compared to the controlled and assisted modes was not confirmed. The intra-group analysis showed an increase in total ventilation in controlled and spontaneous MV mode after using the nebulized bronchodilator, but with different lung mechanic responses. Despite increased ventilation in the dependent pulmonary region, there was no difference in total aeration and ventilation post-nebulization in the assisted MV mode, which could be related to patient–ventilator asynchrony.

## Figures and Tables

**Table 1:** Anthropometric and clinical characteristics.

	Total	PCV (*n* = 5)	A/C PCV (*n* = 7)	PSV (*n* = 7)	*p*-Value
Gender ^a^ male (%)	13 (68.4)	3 (60)	5 (71.4)	5 (71.4)	0.895
Age (years) ^b^	68.6 (13.45)	70.4 (9.65)	67.8 (15.4)	68.1 (15.63)	0.989
ET diameter (mm) ^a^					0.558
7.5 (%)	7 (36.8)	1 (20)	3 (42.9)	3 (42.9)	
8.0 (%)	6 (31.6)	3 (60)	1 (14.3)	2 (28.6)	
8.5 (%)	6 (31.6)	1 (20)	3 (42.9)	2 (28.6)	
Pulmonary disease ^a^ COPD (%) Asthma (%)	17 (89.5) 2 (10.5)	4 (80) 1 (20)	6 (85.7) 1 (14.3)	7 (100) 0 (0)	0.495
Sepsis (%) ^a^	2 (50.2)	0 (0)	1 (14.3)	1 (14.3)	0.671
Duty Cycle (T_I_/T_TOT_) ^b^	0.32 (0.05)	0.31 (0.01)	0.31 (0.21)	0.34 (0.08)	0.535
PEEP (cmH_2_O) ^b^	7.31 (1)	8 (1)	7.14 (1.06)	7 (0.81)	0.231

PCV: pressure-controlled ventilation, A/C PCV: assisted-controlled pressure ventilation, PSV: pressure support ventilation, ET: endotracheal tube, COPD: chronic obstructive pulmonary disease, EIT: electrical impedance tomography, SS: extra small, S: small, M: medium, mm: millimeters, cm: centimeters. ^a^—qualitative variables expressed in absolute and relative frequencies, compared by Chi-squared test. ^b^—quantitative variables expressed as mean and standard deviation, compared by the Kruskal–Wallis test.

**Table 2:** Intergroup analysis of total and ROI ventilation and aeration.

Before	PCV (*n* = 5)	A/C PCV (*n* = 7)	PSV (*n* = 7)	*p*-Value *
V_T_ (L)	0.442 (0.99)	0.373 (0.1)	0.394 (0.1)	0.617
Ti (s)	1.09 (0.14)	1.02 (0.09)	1.07 (0.25)	0.731
∆Z_ND_ (AU)	0.055 (0.02)	0.051 (0.03)	0.048 (0.02)	0.873
∆Z_D_ (AU)	0.046 (0.01)	0.037 (0.01)	0.049 (0.01)	0.183
∆Z_TOT_ (AU)	0.101 (0.03)	0.089 (0.03)	0.098 (0.04)	0.897
EELV_ND_	0	0	0	NS
EELV_D_	0	0	0	NS
EELV_TOT_	0	0	0	NS

PCV: pressure-controlled ventilation, A/C PCV: assisted-controlled pressure ventilation, PSV: pressure support ventilation, **∆**Z_ND_: impedance variation in non-dependent pulmonary region, **∆**Z_D_: impedance variation in dependent pulmonary region, **∆**Z _TOT_: total impedance variation, EELV_ND_: end-expiratory lung volume in non-dependent pulmonary region, EELV_D_: end-expiratory lung volume in dependent pulmonary region, EELV_TOT_: total end-expiratory lung volume, AU: arbitrary unit, L: liters, s: seconds, NS: non significant. * Kruskal–Wallis test.

## Data Availability

The data presented in this study are available on request from the corresponding author. The data are not publicly available due to ethical restrictions.
